# Are we developing walkable suburbs through urban planning policy? Identifying the mix of design requirements to optimise walking outcomes from the ‘Liveable Neighbourhoods’ planning policy in Perth, Western Australia

**DOI:** 10.1186/s12966-015-0225-1

**Published:** 2015-05-16

**Authors:** Paula Hooper, Matthew Knuiman, Fiona Bull, Evan Jones, Billie Giles-Corti

**Affiliations:** Centre for the Built Environment and Health, School of Earth and Environment and School of Sports Science, Exercise and Health, The University of Western Australia, 35 Stirling Highway, Crawley, Perth, WA 6009 Australia; School of Population Health, The University of Western Australia, 35 Stirling Highway, Crawley, Perth, WA 6009 Australia; Acuitus, Level 1, 322 Hay Street, Subiaco, Perth, WA 6008 Australia; McCaughey VicHealth Centre for Community Wellbeing, Melbourne School of Population Health, University of Melbourne Australia, Melbourne, VIC Australia

**Keywords:** Walking, Planning policy, Cluster analysis, Built environment, Evaluation, New urbanism, Liveability health promotion

## Abstract

**Background:**

Planning policy makers and practitioners are requesting clearer guidance on the ‘essential’ ingredients as assessed by public health researchers to ensure suburban neighbourhood environments are designed to promote active living behaviours such as walking.

**Objectives:**

To identify the combination of design requirements from the ‘Liveable Neighbourhoods’ (LN) planning policy in Perth, Western Australia that were optimally supportive of walking.

**Methods:**

K-means cluster analysis identified groups of developments with homogeneous LN features from its community design (CD), movement network (MN), lot layout (LL) and public parkland (PP) elements. Walking behaviours measured using the Neighbourhood Physical Activity Questionnaire were compared between participants resident in the different clusters, adjusting for demographic characteristics, self-selection factors, stage of construction and scale of development.

**Results:**

Compared with participants living in the referent cluster of ‘poor CD and PP developments’ those living in: ‘MN and LL developments’ had higher odds of doing any (OR = 1.74; 95 % CI = 1.22, 2.48) and ≥60 min walking for recreation (WR) (OR = 2.05; 1.46, 2.88); ‘PP developments’ had increased odds of doing any WR (OR = 3.53; 2.02, 6.17), ≥60 min WR (OR = 3.37; 1.98, 5.74) and any total walking (TW) (OR = 2.35; 1.36, 4.09); ‘CD-MN developments’ had increased odds of doing any walking for transport (WT) (OR = 2.64; 1.38, 5.06), ≥60 min WT (OR = 1.98; 1.09, 3.61), any TW (OR = 1.71; 1.44, 2.03), ≥60 min TW (OR = 1.77; 1.14, 2.76) and ≥150 min TW (OR = 1.47; 1.15, 1.86).

**Conclusions:**

This study is the first to have empirically identified a mix of specific and distinguishing planning policy neighbourhood design requirements to optimise walking outcomes. These findings will assist in the assessment of urban plans for greenfield suburban developments designed to promote walking and physical activity.

## Background

Government policy and planning initiatives determine the way cities, towns and neighbourhoods are developed and configured, and are inextricably linked to all aspects of human health and health-related behaviours. They also play a vital role in creating and shaping the environments which support or undermine residents’ ability to be safely and conveniently physically active [1–4]. However, despite a proliferation of research evidence on the associations between the built environment, suburban sprawl and walking [5, 6, 3, 4, 7, 8, 1], it is widely acknowledged that to advance this field, more research is required that assesses the public health dimensions of planning regulations and policies through the evaluation of innovative communities and environmental or planning policies, programs or codes that promote active living [9–11]. Moreover, planning professionals and policy makers have identified the need for more evidence on the types and mix of infrastructure needed to support health and active living behaviours and the effectiveness of planning policies to influence planning practice and policy [12, 13].

In February 1998 the Western Australia State Government identified the need to redress the impacts of conventional development planning policies that had facilitated car dependence and sprawl across Perth to create more sustainable suburban communities, launching the “Liveable Neighbourhoods Community Design Guidelines” (LN) [14–16]. LN is based on the vision and principles of New Urbanism [17, 18] advocating for mixed-use, pedestrian-orientated, compact developments and human-scaled neighbourhoods through four elements - community design (CD), movement networks (MN), lot layout (LL) and public parkland (PP) [15]. A key intended (and specified) outcome of the LN was to reduce car dependence and encourage more active forms of transport in the form of walking, cycling and public transport use [15]. This is now the preferred operational policy of the Western Australian Planning Commission (WAPC) for the design and assessment of structure plans (regional, district and local) and subdivisions, for new urban and suburban (predominantly residential) areas in the metropolitan area, country centres, and on greenfield and large brownfield and urban infill sites. The implementation of LN provided a unique opportunity for an independent evaluation of this ‘natural experiment’ and as described fully elsewhere [19], in 2003, the RESIDential Environments project (RESIDE) was initiated.

Twenty-thirteen marked the fifteenth anniversary of the introduction of LN and the start of a comprehensive review process by the Department of Planning. As a consequence, there has been considerable interest in the RESIDE findings and particularly whether the results might assist in identifying the ‘essential’ combination or mix of design features, from the large number of design features currently stipulated in the LN policy document, to ensure the design of suburban neighbourhood environments promote active living behaviours such as walking and physical activity.

This work has already commenced; for example, as part of the RESIDE study a process evaluation was conducted to measure the policy implementation in 36 housing developments (19 LN and 18 conventional developments) using specifically tailored spatial measures corresponding with the LN design requirements [20]. Results revealed a strong dose–response relationship between policy compliance and walking [20] and that despite incomplete implementation of all LN requirements, for every 10 % increase in compliance, the odds of participants doing any walking for transport within the neighbourhood increased by a factor of 1.53 (1.13-2.08) [20]. Moreover, for every 10 % increment in compliance with the MN, CD and LL elements, the odds of participants doing any walking for transport within the neighbourhood increased by a factor of 2.48 (1.38-4.50), 1.27 (1.13-1.42), and 1.26 (1.06-1.50), respectively [20].

Other researchers have also started to explore the cumulative effects of environmental attributes that might better explain walking [21-23,6,24,25] using cluster analysis. This technique characterises or profiles neighbourhoods based on their multidimensional features allowing co-existing features to be identified. Such approaches have revealed neighbourhood types with different combinations of characteristics that are important for supporting or encouraging physical activity behaviours [21,26].

Our previous analyses examined overall policy compliance and walking outcomes, and found walking for transport and recreation (any and ≥60 min per week) associated with varying levels of compliance with different combinations of LN’s four policy elements [20]. However, each of the four elements within the policy document contains up to 25 design features that contribute to meeting the elements’ objectives. Identifying which of these design features are most important is a frequent question asked by planners and urban designers as they require this level of detail to help identify the design requirements they must prioritise.

Therefore, this paper reports an extension to our cluster analyses undertaken to identify the specific design features that differentiate the clusters of developments from one another. The aim of this paper was to identify the ‘*mix*’ of specific design features within each of the four elements of the LN policy responsible for the differences in the walking behaviours of residents.

## Methods

### RESIDE Project: participants and housing developments

The participant recruitment process and selection of housing developments for the RESIDE study are reported in detail elsewhere [19]. In brief, the RESIDE study population comprised people who purchased land and built houses in 73 new suburban housing developments identified by the Department of Planning – nineteen of which were identified as having been approved under the new LN policy. Participants completed a self-report questionnaire before they moved into their new home and on three subsequent occasions after they relocated. This paper reports the evaluation at the third time point in 2009 reflecting 6 years post commencement of RESIDE. A sub-sample of participants (n = 664) were identified who completed a questionnaire about 36 months after relocating to a new neighbourhood.

### Measuring LN policy implementation

Measures specific to 43 of the LN design features across the four elements were developed and computed in GIS for 36 of the housing developments from the RESIDE project (Table 1). The evaluation was specifically concerned with quantifying the levels of policy implementation within the housing developments selected for inclusion in the RESIDE study. As such, all GIS measures were developed for the housing development and a surrounding 800 m Euclidean buffer. All RESIDE participants located within a given housing development were assigned the same scores for each design feature corresponding to that development. Full details of the policy requirements and developed measures are reported elsewhere [20].

**Table 1:** Objective measures of the community design, movement network, lot layout and public parkland requirements from the Liveable Neighbourhoods policy^a^

COMMUNITY DESIGN
Access to Neighbourhood Centres
▪ Distance to the nearest neighbourhood/town centre^1^
▪ Centre accessible within 400 m (Yes/No)^2^
▪ Centre accessible within 800 m (Yes/No)^2^
▪ Centre accessible within 1600 m (Yes/No)^2^
▪ % of dwellings within 400 m of a centre^3^
▪ % of dwellings within 800 m of a centre^3^
▪ % of dwellings within 1600 m of a centre^3^
Configuration of Neighbourhood centre accessible within 1600 m
▪ Main street or big-box layout
Diversity of Destinations within Neighbourhood Centres
▪ Number of convenience goods stores: *Supermarkets; deli’s; speciality food stores(*i.e.*, butchers, greengrocers, fishmongers); liquor stores and bottle shops; newsagents and confectionary retailers; service station shops*
▪ Number of retail goods stores: *Fashion and apparel stores, footwear and accessories shops; jewellery stores; books, games, music, DVD/video stores; cards, souvenirs and gift stores; personal electronic and telecommunications; variety and discount stores*
▪ Number of general services: *Hair and beauty; banks and finance; personal health (*e.g.*, pharmacies); video/DVD rental; laundry and tailoring*
▪ Number of medical and health care services: *Medical centres; other medical and health services (*e.g.*, dentist, physiotherapist);*
▪ Number of places of worship: *Churches, mosques, temples and synagogues*
▪ *Number of community services and facilities: Community centres; day care centres/crèches; libraries*
▪ Number of eating and drinking out establishments: *Restaurants, bars, fast food outlets, hotels, taverns, pubs, bars, nightclubs*
▪ Number of entertainment and amusement places: *Cinemas; theatres; convert halls; museums, art galleries; gaming and gambling venues; sporting (spectator) venues*
▪ Destination diversity score - number of different destination types present within the centre
▪ Minimum uses present within the centre (small retail or convenience store + post box + bus stop) (Yes/No)
Access to Public Transport
▪ Distance to the nearest bus stop^1^
▪ Bus stop accessible within 400 m (Yes/No)^2^
▪ % of dwellings ≤400 m of a bus stop^3^
▪ Number of bus routes through the development
▪ Number of bus services to/from the development
▪ Bus stop accessible within 250 m walkable catchment of the centre (Yes/No) ^2^
▪ Number of bus services to the centre
▪ Distance to the nearest train station^1^
▪ % of dwellings ≤800 m of a train station^3^
▪ % of dwellings ≤1600 m of a train station^3^
Access to Primary Schools
▪ Distance to the nearest primary school^1^
▪ % of dwellings ≤400 m of a primary school^3^
▪ % of dwellings ≤800 m of a primary school^3^
▪ % of dwellings ≤1600 m of a primary school^3^
MOVEMENT NETWORK
Connectivity of the Street Networks
▪ Connected node ratio (number of 3 + 4 way intersections ÷ number of all intersections including culs-de-sac)
▪ Mean block perimeter
Median block perimeter
▪ Block density = number of blocks ÷ constructed land area of development
▪ Walkable block ratio = number of blocks ≤620 m perimeter ÷ total number of blocks
External Connectivity
▪ Number of pedestrian-friendly access points along the development perimeter ÷ perimeter of development boundary (km)
Culs-de-sac Provision and Design
▪ Cul-de-sac length ratio (number of culs-de-sac ≤120 m in length ÷ total number of culs-de-sac)
▪ Cul-de-sac link ratio (number of culs-de-sac with a pedestrian cut through ÷ total number of culs-de-sac)
▪ Cul-de-sac lot ratio (number of culs-de-sac serving ≤20 residential lots ÷ total number of culs-de-sac)
▪ Percentage of residential lots on culs-de-sac (≤ / > 15 %) = number of residential lots served by a culs-de-sac ÷ total number of residential lots)
▪ Culs-de-sac street % (length of all road network segments terminating in a cul-de-sac ÷ total length of all road centrelines)
Total footpath provision
▪ Footpath length per unit area (ha) = length of all footpaths ÷ constructed land area of housing development
▪ Footpath to road ratio = length of all footpaths within the development ÷ length of all roads within the development
Footpaths on both sides of the street?
▪ % of road length with sidewalks (i.e., footpath segments that ran alongside the road)
▪ Sidewalk to road ratio = length of all footpath segments alongside/adjacent to roads ÷ length of all roads
Footpaths within neighbourhood centre 400 m service areas
▪ % of road length with sidewalks (i.e., footpath segments that ran alongside the road)
▪ Sidewalk to road ratio = length of all footpaths alongside roads ÷ length of all roads
Footpaths within primary school 400 m service areas
▪ % of road length with sidewalks (i.e., footpath segments that ran alongside the road)
▪ Sidewalk to road ratio = length of all footpaths alongside roads ÷ length of all roads
Cycling networks
▪ Cyclable roads ratio (based upon the level of stress experienced by the rider as a result of the traffic volumes and speed) = length of low + moderate stress roads (cycling friendly roads) ÷ length of all roads
▪ Cycle path length per unit area (ha) = length of all designated cycle and shared paths ÷ constructed land area of housing development
▪ Cycle path to road ratio = length of all footpaths ÷ length of all roads within the development
Streetscapes – Trees along footpaths
▪ Tree density along footpaths = number of trees along footpaths (within a 5 m buffer) ÷ length (km) of footpaths within the development
▪ Tree canopy cover = are of footpath shaded by tree canopy cover ÷ total footpath area within the development
LOT LAYOUT
Residential lot size
▪ Mean residential lot size
▪ Median residential lot size
▪ Number of different lot sizes present (categories: ≤350 m^2^; >350 - ≤550 m^2^; >550 - ≤750 m^2^; >750 - ≤950 m^2^; >950 m^2^)
▪ Residential land areas occupied by different lot sizes
▪ % of lots ≤350 m^2^ (i.e., “small” lots for medium density housing)
Lots near neighbourhood centres (within 400 m service areas)
▪ Mean residential lot size
▪ Median residential lot size
▪ Number of different lot sizes (categories) present
▪ Residential land area occupied small lots (≤350 m^2^)
Housing diversity development-wide
▪ Number of dwellings by type (n = 9) as a % of the total number of dwellings
▪ Residential land area occupied by different (n = 9) dwelling types
Dwelling types near neighbourhood centres (within 400 m service areas)
▪ Number of dwellings by type (*n = 9*) as a % of the total number of dwellings
▪ Residential land area occupied by different (*n = 9*) dwelling types
*Nine dwelling type categories: 1) Single detached houses; 2) Semi-detached houses; 3) Duplex unit; 4) Triplex unit; 5) Town house; 6) Terrace house; 7) Group house; 8) Villa house; and 9) Flat or apartment. Housing types 3–8 (inclusive) represent medium density housing models. The total number of different dwelling types present within each development was then identified (*i.e. *1–9).*
PUBLIC PARKLAND
Amount and type of parks
▪ Area (ha) of all parks
▪ Area (ha) of all publicly accessible school grounds
▪ % provision of parks:
o Percentage park provision = area of all parks ÷ gross constructed land area of housing development (< / ≥ 10 %)
o Percentage park and school grounds provision = area of all parks + publicly accessible school grounds ÷ gross constructed land area of housing development (< / ≥ 10 %)
▪ Area of local parks types as a % of the total parkland area
▪ Area of neighborhood parks types as a % of the total parkland area
▪ Area of district parks types as a % of the total parkland area
Access to parks
▪ Distance to the nearest park (of any size)^1^
▪ Distance to the nearest local park^1^
▪ Distance to the nearest neighborhood park^1^
▪ Distance to the nearest district park^1^
▪ Distance to the nearest regional park (>4 ha)^1^
▪ % dwellings ≤400 m of any park^3^
▪ % dwellings ≤200 m of a local park^3^
▪ % dwellings ≤400 m of a small neighborhood park^3^
▪ % dwellings ≤400 m of a medium neighborhood park^3^
▪ % dwellings ≤400 m of a large neighborhood park^3^
▪ % dwellings ≤600 m of a district park^3^
% dwellings ≤2.5 km of a regional-sized park (>4 ha)^3^
▪ Park perimeter frontage ratio = % of the park perimeter bordered by lots facing the park
▪ Park perimeter roads ratio = % of the park perimeter bordered by adjacent roads

^*a*^ *This is an abridged version of a Table previously published by the authors* [20]

^1^Distance computed along the road network from all residential dwelling points (n = 31,102) to the nearest centre, bus stop, train station, primary school and parks. For each development the mean distance to each of these destinations was computed

^2^Deemed accessible if ≥10 % of the dwellings within a development had access to a centre within the specified distance

^3^Number of residential dwellings within a housing development that were within the specified distance (along the road network) as a proportion of the total number of residential dwellings within that development

### Measurement of walking behaviours

Self-reported walking behaviors were measured using the ‘Neighbourhood Physical Activity Questionnaire’ (NPAQ) [27]. Participants reported the frequency and duration of walking for transport (WT) and recreation (WR) within their neighbourhood (defined as a 1.6 km service area or 10 to 15 min walk from home) in a usual week [27]. Seven walking outcomes were analysed: four dichotomous variables (yes/no) were computed for >0 min (any) and ≥60 min per week of any walking WR and WT in the neighbourhood as per previous analyses [20]. For the purposes of this study minutes of total walking (TW) were also dichotomised (yes/no) at >0 min (any), ≥60 min and ≥ 150 min per week (i.e., meeting the global recommended levels of physical activity through any type of walking) [28].

### Identifying cluster-derived development types

Cluster analysis is an ‘exploratory data analytical tool for organising observed data into meaningful groups based on combinations of independent variables which maximises the similarity of cases within each group while maximising the dissimilarity between groups that are initially unknown’ [29, 30]. The k-means method is the most common of the ‘partitioning’ clustering analyses and is generally thought to be superior to hierarchical methods as it is less affected by outliers and the presence of irrelevant clustering variables [30].

A series of k-means cluster analyses were run on all 43 measured design features to identify clusters of developments that were homogeneous with respect to their implementation of the various design features. Prior to running the cluster analysis, all of the variables were standardised (i.e., z-scores). A number of cluster solutions were obtained and the within-cluster variance was used to decide on the optimal number of clusters (i.e., the number at which any further increase in clusters produced only a marginal reduction in the within-cluster variance). Once the clusters were determined (*n* = 4 clusters), and for the purposes of this study, a one-way ANOVA was run on the z-scores of the raw variables (i.e., design features) to assess how statistically distinct the different clusters were from one another and which classifying variables were significantly different between the cluster groupings; that is which of the policy’s design features contributed most to the cluster solution. Tukey’s post-hoc tests were run to determine where any significant differences lay and assist in determining the differentiating characteristics of the different clusters.

### Data analysis

All participants were assigned the cluster number of the housing development in which they were resident. The level of walking of participants resident in the different clusters was compared using logistic regression models with generalised estimating equations (GEE) that allowed for correlations between participants within the same development and that controlled for the following: demographic variables (age; gender; education, children ≤18 years and under living at home); stage of construction (i.e., the percentage of the development land area that had been constructed at the time of evaluation); size of development (i.e., subdivisions <100 ha, structure plans 100-300 ha or regional developments >300 ha); and self-selection factors. To measure self-selection, the RESIDE baseline survey asked participants to rank the importance of 21 factors influencing their choice of new housing development and 12 of these matched the LN design features and were used in analyses to control for residents’ preferences.

## Results

Full details of the socio-demographic characteristics of this sub sample of the RESIDE participants have been reported elsewhere [20]. Briefly, the average age of participants was 43 years (SD 11.7), the majority were female (62 %), married or in a de-facto relationship (87 %) and about one-half of participants had one or more children under the age of 18 years living at home. There was no significant difference between the four clusters on any of the socio-demographic variables. However, there were clear differences between the cluster groups on the level of compliance of the four LN elements and implementation of the 43 design features.

The labelling of each cluster attempts to capture the mix and levels of implementation of the design features from the four elements (Table 2) and the subsequent experience of living in neighbourhoods with these features (or lack of particular features).

**Table 2:** Coding of cluster of developments based on the levels of implementation and mix of policy design features

Cluster Type	Movement Network	Lot Layout	Public Parkland	Community Design
Disconnected Developments	Rank 4^th^ = worst	Rank 4^th^ = worst	Rank 4^th^ = worst	Rank 4^th^ = worst
Connected and Compacted Developments	Rank 1^st^ = best	Rank 1^st^ = best	Rank 3^rd^	Rank 3^rd^
Green Developments	Rank 3^rd^	Rank 3^rd^	Rank 1^st^ = best	Rank 2^nd^
Liveable Developments	Rank 2^nd^	Rank 2^nd^	Rank 2^nd^	Rank 1^st^ = best

**Fig. 1:**
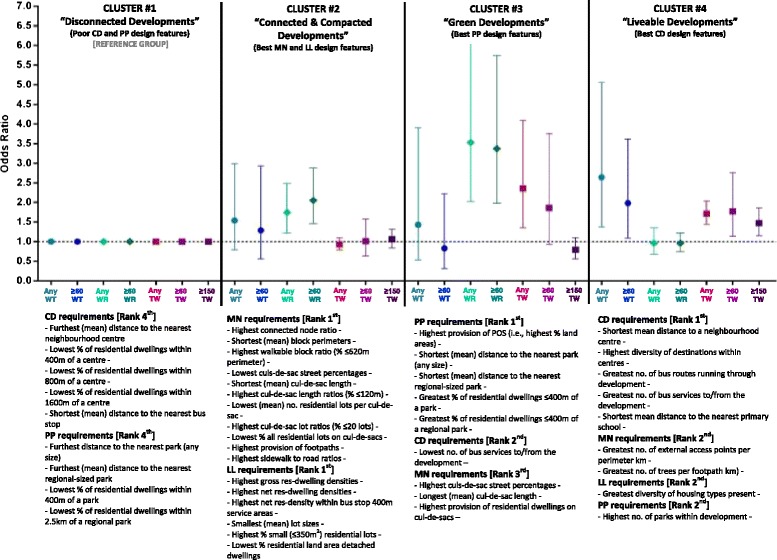
Cluster-derived development types and the key mix of Liveable Neighbourhoods policy design features and associations with walking behaviours

Figure 1 presents the distinguishing characteristics (i.e., design features) for each of the four clusters of development types identified from the ANOVAs. It also presents the odds ratio’s and 95 % confidence intervals for walking behaviours of the RESIDE participants resident in each of the clustered development types, in comparison to residents in the referent development, Cluster #1.

Cluster #1 – the *‘disconnected developments’* comprised five developments (n = 74 participants) that performed poorly (compared with the three other clusters) in terms of compliance of the four LN policy elements. It was characterised by having the poorest implementation of the CD and PP design features, with the worst accessibility (in terms of average distances from all residential lots and the proportion of dwellings within the policy stipulated distances) to neighbourhood centres and areas of public open space (Fig. 1). Hence, the *‘disconnected developments’* cluster was selected as the reference group of developments to which all other clusters were compared.

Cluster #2 – the *‘connected and compacted developments’* comprised 23 developments (n = 436 participants) and was characterised as having the best levels of compliance with the MN and LL elements. This group of developments outperformed the others in terms of their implementation of the majority of requirements for the MN element. The distinguishing characteristics of the movement networks in these developments were smaller block sizes, shorter and more connected culs-de-sac and high provisions of footpath infrastructure (Fig. 1). These developments also had the highest levels of compliance with LL element and specifically requirements for residential density and provision of small lots capable of supporting medium density housing (Fig. 1). Compared with the *‘disconnected developments’* cluster, participants resident in these developments had significantly increased odds of doing any WR (OR = 1.74 1.22, 2.48) and ≥60 min of WR (2.05 1.46, 2.88) in a usual week.

Cluster #3 – the *‘green developments’* comprised five developments (n = 47 participants) and were characterised as having the highest levels of compliance with the PP element and the worst levels of public transport provision and services to/from the developments. These developments significantly outperformed the others in terms of its provision of POS and access to POS (as measured by the greatest proportion of residential dwellings within 400 m of any park and 2.5 km of a regional-sized park ≥4 ha). These developments also performed relatively well on the implementation of the MN requirements but were characterised as having more and longer culs-de-sac. Compared with those in the *‘disconnected developments’* cluster, participants resident in the *‘green developments’* had significantly increased odds of doing any WR (OR = 3.53 2.02, 6.17) and ≥60 min of WR (OR = 3.37 1.98, 5.74) in a usual week. The odds of doing ≥150 min of WR was also increased and approaching significance (OR = 1.98 0.92, 4.29 *p* = 0.080; results not shown). The presence of these features was also associated with increased odds of doing any TW (OR = 2.35 1.36, 4.09). The odds of participants in these developments doing ≥60 min TW was also approaching significance (OR = 1.86 0.93, 3.75, *p* = 0.081).

Cluster #4 – the *‘liveable developments’* comprised three developments (n = 107 participants). These developments had the best levels of compliance with the CD element and requirements related to access to neighbourhood centres and the diversity of destinations within the town centres which included a mix of supermarkets and convenience shops, daily general services (e.g., banks and pharmacies), health, welfare and community services (e.g., child care, community and medical centres), as well social destinations such as cafes, restaurants and pubs or taverns). They were also served by the greatest number of bus routes and services and had the highest number of parks present (Fig. 1). These developments also performed reasonably well on the implementation of the movement network requirements and in particular they had the best levels of external connectivity (i.e., the greatest number of external access points per km along the development perimeter) and the best provision of trees along the footpath networks. Compared with those in the *‘disconnected developments’* cluster, participants resident in these developments had significantly increased odds of doing any WT (OR = 2.64 1.38, 5.06) and ≥60 min of WT (OR = 1.98 1.09, 3.61) in a usual week. The presence of these features was also associated with increased odds of doing any TW (OR = 1.71 1.44, 2.03), ≥60 min TW (OR = 1.77 1.14, 2.76) and ≥150 min TW (OR = 1.47 1.15, 1.86).

## Discussion

This paper reports new results on the evaluation of an operational State planning policy (‘Liveable Neighbourhoods’) in Perth, Western Australia. By further unpacking our previous cluster analyses we were able to identify which of the 43 specific design features differentiated the clusters of developments that contributed to more walking. This analytical approach aimed to help determine the mix of key policy requirements most important for encouraging and supporting walking behaviours, out of a very large number of policy requirements.

Using cluster analysis four distinct groups of housing developments were identified based on implementation of the LN requirements. The odds of those participants living in the ‘connected and compacted developments’ doing any and 60 min or more per week of recreational walking was double compared to those living in the *‘disconnected developments’*. These developments out-performed others in implementing the movement network and lot layout requirements but provided limited access to neighbourhood centres or parks. Residents living in the ‘Green developments’ however had three times greater odds of doing any and 60 min or more per week of recreational walking compared with those living in the *‘disconnected developments’* developments. Finally, the odds of doing some and 60 min or more per week of transport-related walking for those living in the ‘best CD developments’ were double that of those living in the *‘disconnected developments’*. These main results are considered in more detail.

### What LN requirements encouraged recreational walking?

An important determinant of whether people walk locally is the presence and continuity of pedestrian infrastructure (i.e., footpaths or sidewalks); these features were well provided in the *‘connected and compacted developments’*. However, the majority of these developments were smaller residential subdivision developments, and as such, too small to warrant the inclusion of a neighbourhood centre. But, this omission means that they fail to provide a ‘complete neighbourhood’ as envisaged by the LN policy [15]. When smaller residential developments or subdivisions (often undertaken as part of urban infill) are approved in an *unplanned* manner it is difficult to ensure that shops, services, places of work, community facilities and POS are provided and distributed equitably and within walking distances. This can result in widespread disjointed residential developments, none of which is large enough to warrant the provision of centres and other important community infrastructures [31] leaving residents of these subdivisions with few (if any) destinations within walking distance. This explains why the levels and types of walking observed in the *‘connected and compacted developments’* was mainly recreational, rather than transport-related. This observation is supported by a UK study that also found that *ad-hoc* small developments had limited effects on travel behaviour [32]. Both studies and others highlight the need for planning at the neighbourhood scale to provide proximal destinations to which people can walk [33, 8].

Participants resident in the *‘green developments’* had the best provision of, and access to POS delivered in accordance with the LN policy (i.e., ≥8 % of the gross subdivisible land area), and had higher odds of doing any and ≥60 min of weekly walking for recreation; in fact over three times greater odds of walking for recreation than those living in the *‘connected and compacted developments’* which are characterised by only providing a good supportive movement network. The likelihood of meeting recommended levels of physical activity through recreational walking was also higher and approached significance (OR = 1.98, 0.92, 4.29, *p* = 0.080; these results not shown earlier). Compliance with POS provisions also increased the likelihood of doing any and ≥60 min of total walking by approximately 86 % and this approached significance (OR = 1.86 0.93, 3.75 *p* = 0.081). A large proportion of the total walking by participants in Cluster #3 (the *‘green developments’*) is likely to be accounted for by recreational walking, rather than transport walking. This finding is supported by our longitudinal analyses that showed for each type of recreational destination (i.e., park, playing field or beach) gained after relocation participants recreational-walking increased by around 18 min/week [3].

The LN policy calls for the provision of a range of open space types (i.e., sizes) to meet the needs of all users groups [15] and a balance between smaller local (≤0.3 ha) and neighbourhood parks (>0.3-2.5 ha) and larger district playing fields (≥2.5-7 ha). It is suggested that the former are provided within short walking distances from home and distributed along a connected footpath network providing spaces for small playgrounds, meeting and resting place. Larger playing fields should be shared between neighbourhoods and capable of accommodating formal sports activities and active recreational pursuits as well as a destination for people to walk to, in, and around. Whilst our previous findings found that compliance with the public parkland element alone was not associated with levels of walking for recreation [20], the results of this cluster analysis suggest that it is the implementation of the parkland design features *in combination* with the provision of connected street networks and footpath infrastructure (as intended by the policy) that is necessary to support local recreational walking. This finding is consistent with previous evidence that has identified associations between walking for recreation and the provision of larger, attractive parks as destinations for people to walk to and in or around [34]. However, aesthetic features of streetscapes have also been associated with walking for recreation [35]. As such the provision of smaller parks distributed along connected footpath networks may enhance neighbourhood greenness and aesthetics making neighbourhoods more attractive and enjoyable for recreational walking [3, 7]. More work is needed to explain the role and contribution of different sized parks in supporting recreational walking to tease out their importance as an alternative or addition to larger parks. Given the additional cost of maintaining many smaller, rather than fewer larger parks, this type of information is important for resource-constrained local governments which are responsible for park maintenance.

Parks can be destinations to walk to, however Moudon and colleagues [36] found that whilst parks and areas of open space were important for physical activity, they do not provide anchor points in walkable neighbourhoods. Rather, “attractor destinations” for walkable neighbourhoods are centred on daily retail and food-related activities associated with necessary rather than discretionary spending [36]. Other studies have also found that public open space may serve as a significant focal point or destination for leisure-related or recreational walking but less so for transport-related walking trips [37]. These studies combined with our own findings reinforce that other factors are associated with walking for transport; these are now considered.

### What LN requirements encouraged transport-walking?

Previous findings have shown that compliance with the MN element is associated with the largest odds of walking for transport, but after adjustment in a multivariate model that included all four LN elements, the CD element remained the most important for walking for transport [20]. The intent of the community design element is to create suburban environments that provide for residents’ diverse daily needs through the development of walkable local neighbourhood or town centres which act as community focal points or hubs, with a concentration of destinations and mixed land uses that attract people for a variety of activities [15]. These are vital to support local walking as a part of daily routine and providing people with the option to live in new areas without the need for a car. Hence, residents in the ‘best CD developments’ had the highest odds of doing any and 60 min or more of walking for transport in a usual week. They were also 50 % more likely to meet recommended levels of physical activity in a usual week (≥150 min) through walking in their neighbourhood. Residents of these developments had the best access to neighbourhood centres with the greatest diversity of destinations as envisaged by LN. These developments also had reasonable implementation of the movement network requirements providing particularly high levels of external connectivity and provision of trees along the footpath networks. Trees have been shown to be supportive features for walking in the neighbourhood [7, 8].

### Comprehensive New Urbanist principles are essential

The LN guidelines are based on New Urbanist principles. These results from a novel application of cluster analysis provide robust empirical evidence that communities built in accordance with these principles, will be likely to increase physical activity through walking. However, these results also highlight the importance of implementing the combination of elements required to support positive outcomes. Importantly, we found that compliance with the movement network and lot layout elements was essential to provide the framework of, and to underpin, a walkable community: that is, to ensure the provision of compact, well connected street networks with adequate pedestrian infrastructure. However, above and beyond these design features, the added combination of implementation of the community design and public parkland elements is critical to ensure residents have access to a mix of recreational and utilitarian destinations to which to walk, thereby supporting walking locally for both recreation and transport.

The ability of neighbourhoods to provide the full suite of social infrastructure required to create liveable pedestrian-friendly developments depends on the size of the development. Whilst LN sets out a comprehensive approach to the regional structuring of walkable neighbourhoods clustered to form towns with interconnected streets, its application at smaller subdivision development levels, such as those in cluster #2, simply do not have enough land area to provide all essential community infrastructure. Ensuring that new housing developments are cohesive with the wider environment and with the walking, cycling and public transport movement networks and community facilities and destinations is difficult to achieve when adjacent developments are managed by different developers and are built at different times (and according to different planning requirements). Therefore, the development of smaller subdivisions should be regionally planned with their development co-ordinated to ensure timely and equitable provision of shops and services within walking distances. Local government planning schemes should specifically map out and identify the locations of essential community design facilities, such as schools, neighbourhood centres, social infrastructure and services and POS, as well as pedestrian and bicycle networks. The schemes should identify the responsibilities and contributions required from the state, local governments and private developers and where and what private developers need to contribute towards the installation of these infrastructure and facilities. This could be achieved through the pooling of land contributions from adjacent developments for POS or neighbourhood centres and other social infrastructure.

The study was conducted in new suburban residential environments, typically middle to higher socio-economically rated neighbourhoods. Consequently, the findings may represent a middle-class suburban phenomenon of new home buyers moving into new housing developments located on the urban fringe. However, this was unavoidable given RESIDE’s main strength which was to study the impact of a planning policy intended to improve the built form of such suburban greenfield developments. Whilst the findings may be less transferable to other settings (i.e. higher density urban and rural areas or settings) or populations (i.e., lower SES) the results are highly applicable to many urban and suburban areas throughout Australia and the United States (US). Moreover, many new developments on the urban fringe provide more affordable housing to lower income households. In addition, as new developments evolve and rental housing becomes more available, the results are likely to apply to these residents, as much as home owners, even though given our study design, these residents were not included in our study. Self-administered physical activity instruments also have well documented limitations [38], however, the NPAQ has previously been shown to have acceptable reliability [27] and was designed specifically to evaluate urban planning policies designed to encourage local walking. Apart from limitations, this study has several strengths. It is unique in that its environmental measurements included those from an operational planning policy and it empirically identified a mix of specific and distinguishing planning policy design requirements to optimise walking outcomes. It therefore contributes to a known gap in the evidence-base [39].

Figure 1 identifies the mix of design features from an operational planning policy that supported local walking. These have the potential to form the basis of a set of critical design features of key performance indicators (KPIs) for the LN policy and other similar planning schemes. This will assist in the assessment and approvals of designs for health-enhancing greenfield suburban environments to promote walking and physical activity. Further research could extend our analyses to test whether our findings are equally relevant in other jurisdictions. Additional research is also needed to identify the optimal amount of infrastructure required to *optimise* walking. These results could inform whether current policy LN design features could align, and inform future policy directions.

## Abbreviations

CD: Community design
GIS: Geographic information system
LN: Liveable neighbourhoods community design guide planning policy
LL: Lot layout
MN: Movement network
PP: Public parkland
POS: Public open space
TW: Total walking
WR: Walking for recreation
WT: Walking for transport

## References

[1] Transportation Research Board Institute of Medicine (2005). Does the Built Environment Influence Physical Activity? Examining the Evidence.

[2] Witten K, Hiscock R, Pearce J, Blakely T (2008). Neighbourhood access to open spaces and physical activity of residents: A national study. Prev Med.

[3] Giles-Corti B, Bull F, Knuiman M, McCormack G, Van Niel K, Timperio A (2013). The influence of urban design on neighbourhood walking following residential relocation: Longitudinal results from the RESIDE study. Soc Sci Med.

[4] Knuiman MW, Christian HE, Divitini ML, Foster SA, Bull FC, Badland HM (2014). A Longitudinal Analysis of the Influence of the Neighborhood Built Environment on Walking for Transportation: The RESIDE Study. Am J Epidemiol.

[5] Frank L, Andresen M, Schmid T (2004). Obesity Relationships with Community Design, Physical Activity and Time Spent in Cars. Am J Prev Med.

[6] Frank L, Saelens B, Powell K, Chapman J (2007). Stepping towards causation: Do built environments or neighborhood and travel preferences explain physical activity, driving and obesity?. Soc Sci Med.

[7] Owen N, Humpel N, Leslie E, Bauman A, Sallis J (2004). Understanding Environmental Influences on Walking: Review and Research Agenda. Am J Prev Med.

[8] Saelens B, Handy S (2008). Built Environment Correlates of Walking: A Review. Med Sci Sports Exerc.

[9] Ogilvie D, Charles EF, Helen R, Nick C, Val H, Claire FF (2007). Interventions to promote walking: systematic review. BMJ.

[10] Sallis J, Bauman A, Pratt M (1998). Environmental and Policy Interventions to Promote Physical Activity. Am J Prev Med.

[11] Schilling J, Linton LS (2005). The Public Health Roots of Zoning: In Search of Active Living’s Legal Genealogy. Am J Prev Med.

[12] Allender S, Cavill N, Parker M, Foster C (2009). ‘Tell us something we don’t already know or do!’ The response of planning and transport professionals to public health guidance on the built environment and physical activity. J Public Health Policy.

[13] Koohsari MJ, Badland H, Giles-Corti B (2013). (Re)Designing the built environment to support physical activity: Bringing public health back into urban design and planning. Cities.

[14] Western Australian Planning Commission (1997). Liveable Neighbourhoods Community Design Code: A Western Australian Government Sustainable Cities Initiative. Edition 1.

[15] Western Australian Planning Commission (2002). Liveable Neighbourhoods Community Design Code: A Western Australian Government Sustainable Cities Initiative. Edition 2.

[16] Western Australian Planning Commission (2007). Liveable Neighbourhoods Community Design Code: A Western Australian Government Sustainable Cities Initiative. Edition 4.

[17] CNU. Congress for the New Urbanism. Congress for the New Urbanism,. 1997. http://www.cnu.org/. Accessed 24 September 2010.

[18] Duany A, Plater-Zyberk E, Speck J (2000). Suburban Nation: The Rise of Sprawl and the Decline of the American Dream.

[19] Giles-Corti B, Knuiman M, Timperio A, Van Niel K, Pikora T, Bull F (2008). Evaluation of the implementation of a state government community design policy aimed at increasing local walking: Design issues and baseline results from RESIDE, Perth Western Australia. Prev Med.

[20] Hooper P, Giles-Corti B, Knuiman M (2014). Evaluating the implementation and active living impacts of a state government planning policy designed to create walkable neighborhoods in Perth, Western Australia. Am J Health Promot.

[21] Adams MA, Sallis JF, Kerr J, Conway TL, Saelens BE, Frank LD (2011). Neighborhood environment profiles related to physical activity and weight status: A latent profile analysis. Prev Med.

[22] Cerin E, Leslie E, du Toit L, Owen N, Frank L (2007). Destinations that matter: Associations with walking for transport. Health Place.

[23] Charreire H, Weber C, Chaix B, Salze P, Casey R, Banos A (2012). Identifying built environmental patterns using cluster analysis and GIS: Relationships with walking, cycling and body mass index in french adults. Int J Behav Nutr Phys Act.

[24] McNally M, Kulkarni A (1999). An assessment of the interaction of land use-transportation system and travel behaviour. Transp Res Rec.

[25] McCormack GR, Friedenreich C, Sandalack BA, Giles-Corti B, Doyle-Baker PK, Shiell A (2012). The relationship between cluster-analysis derived walkability and local recreational and transportation walking among Canadian adults. Health Place.

[26] Nelson MC, Gordon-Larsen P, Song Y, Popkin BM (2006). Built and Social Environments: Associations with Adolescent Overweight and Activity. Am J Prev Med.

[27] Giles-Corti B, Timperio A, Cutt H, Pikora TJ, Bull FCL, Knuiman M (2006). Development of a reliable measure of walking within and outside the local neighborhood: RESIDE’s Neighborhood Physical Activity Questionnaire. Prev Med.

[28] World Health Organization (2010). Global recommendations on physical activity for health.

[29] Bailey K, Uprichard E (2012). Cluster Analysis. Byrne D.

[30] Mooi E, Mooi E, Sarstedt M (2011). Cluster Analysis. A Concise Guide to Market Research: The Process, Data and Methods Using IBM SPSS Statistics.

[31] Southworth M (1997). Walkable Suburbs?: An Evaluation of Neotraditional Communities at the Urban Edge. J Am Plan Assoc.

[32] Williams K, Willams K, Burton E, Jenks M (2001). Does intensifying cities make them more sustainable?. Achieving Sustainable Urban Form.

[33] McCormack GR, Giles-Corti B, Bulsara M (2008). The relationship between destination proximity, destination mix and physical activity behaviors. Prev Med.

[34] Sugiyama T, Francis J, Middleton NJ, Owen N, Giles-Corti B (2010). Associations Between Recreational Walking and Attractiveness, Size, and Proximity of Neighborhood Open Spaces. Am J Public Health.

[35] Pikora T, Giles-Corti B, Knuiman M, Bull F, Jamrozik K, Donovan R (2006). Neighborhood Environmental Factors Correlated with Walking near Home: Using SPACES. Med Sci Sports Exerc.

[36] Moudon A, Lee C, Cheadle A, Garvin C, Johnson D, Schmid T (2006). Operational Definitions of Walkable Neighborhood: Theoretical and Empirical Insights. J Phys Act Health.

[37] McCormack GR, Rock M, Toohey AM, Hignell D (2010). Characteristics of urban parks associated with park use and physical activity: A review of qualitative research. Health Place.

[38] Sallis JF, Saelens B (2000). Assessment of physical actiivty by self-report: status, limitations and future directions. Res Q Exerc Sport.

[39] Durand CP, Andalib M, Dunton GF, Wolch J, Pentz MA (2011). A systematic review of built environment factors related to physical activity and obesity risk: implications for smart growth urban planning. Obes Rev.

